# Statistical analysis of *post mortem *DNA damage-derived miscoding lesions in Neandertal mitochondrial DNA

**DOI:** 10.1186/1756-0500-1-40

**Published:** 2008-07-10

**Authors:** Sergi Vives, M Thomas Gilbert, Conchita Arenas, Elena Gigli, Oscar Lao, Carles Lalueza-Fox

**Affiliations:** 1 Departament d'Estadística, Facultat de Biologia, Universitat de Barcelona, Avda. Diagonal 645, 08028 Barcelona, Spain; 2 Statistics Department, Columbia University, 1255 Amsterdam Avenue, New York, NY 10027, USA; 3 Department of Biology, Center for Ancient Genetics, Universitetsparken 15, DK-2100 Copenhagen Ø, Denmark; 4 Institut de Biologia Evolutiva (CSIC-UPF), Dr. Aiguader 88, 08003 Barcelona, Spain; 5 Department of Forensic Molecular Biology, Erasmus Medical Center Rotterdam, Rotterdam, The Netherlands

## Abstract

**Background:**

We have analysed the distribution of *post mortem *DNA damage derived miscoding lesions from the datasets of seven published Neandertal specimens that have extensive cloned sequence coverage over the mitochondrial DNA (mtDNA) hypervariable region 1 (HVS1). The analysis was restricted to C→T and G→A miscoding lesions (the predominant manifestation of *post mortem *damage) that are seen at a frequency of more than one clone among sequences from a single PCR, but do not represent the true endogenous sequence.

**Findings:**

The data indicates an extreme bias towards C→T over G→A miscoding lesions (observed ratio of 67:2 compared to an expected ratio of 7:2), implying that the mtDNA Light strand molecule suffers proportionally more damage-derived miscoding lesions than the Heavy strand.

**Conclusion:**

The clustering of Cs in the Light strand as opposed to the singleton pattern of Cs in the Heavy strand could explain the observed bias, a phenomenon that could be further tested with non-PCR based approaches. The characterization of the HVS1 *hotspots *will be of use to future Neandertal mtDNA studies, with specific regards to assessing the authenticity of new positions previously unknown to be polymorphic.

## Findings

The retrieval of DNA from extinct humans such as Neandertals is technically challenged by problems associated with *post mortem *damage of the original DNA [1]. The growing availability of Neandertal mitochondrial DNA (mtDNA) hypervariable (HVS) sequences (predominantly HVS1), generated with the polymerase chain reaction (PCR) provides a novel dataset to study miscoding lesions associated to DNA damage.

The identification of true *post mortem *damage-derived miscoding lesions in ancient DNA studies, and their discrimination from other PCR artifacts, has been subject of much debate. Although the predominant cause was originally argued to be due to cytosine deamination, generating C→T and G→A miscoding lesions in the retrieved sequences [2,3], a number of studies that examined additional datasets suggested that damage may also include adenine to hypoxanthine modifications, thus resulting in A→G and T→C miscoding lesions [4,5]. The advent of 454/FLX sequencing technology, that allows the identification of which single DNA strand has been sequenced, has helped resolve this debate. In agreement with the original hypotheses [2,3], it is now generally accepted that cytosine deamination is the sole cause of damage-derived miscoding lesions, observed as C→T or G→A miscoding lesions [6-9].

We have investigated the distribution of *post mortem *damage-derived C→T and G→A miscoding lesions in a dataset of Neanderthal HVS1 cloned PCR products. To discriminate between true damage and other PCR artifacts, we took into account only those mutations that are observed as 'consistent' within the datasets, i.e., those base modifications that are observed at a frequency >1 within sequences of a single PCR, but do not represent the consensus sequence as determined through the analysis of multiple independent PCRs of the region [3]. We note that it cannot be assumed, that all the C→T and G→A changes are authentic miscoding lesions, and our analysis likely overestimates the true level as some C→T and G→A changes might be PCR-generated artifacts [9,10].

To exclude other potential biases that might affect the findings, the analysis was furthermore limited to Neandertal sequences that contained the complete Neandertal motif for the amplicon. In this way we were able to exclude contaminant AMH sequences, Neandertal-AMH hybrid sequences, or other artifacts that might derive from jumping-PCR/PCR recombination. As a result of these criteria, the data represents a conservative estimate of the true damage. The goal of the present study is to characterize the different DNA miscoding lesions detected in Neandertals in relation to each specific strand and also to the nucleotide composition. We have also investigated whether the damage is randomly distributed along the HVS1 region, or if there are specific nucleotide positions (sites) that exhibit above expected levels of DNA mutations (termed here *hotspots*). If such miscoding lesion *hotspots *do exist in the Neandertal HVS1 region, then it would be useful to identify them for future Neandertal mtDNA studies, with specific regards to the authentication of new positions previously unknown to be polymorphic in Neandertals.

## Methods

The cloned sequences from the HVS1 fragment of the mitochondrial DNA (mtDNA) of the seven Neandertal specimens that exist with extensive (>300 nucleotides) coverage were used in the analysis. These include: Feldhofer 1 and 2 from Germany [11,12], Mezmaiskaya from Russia [13], Vindija 80 from Croatia [14], Monti Lessini from Italy [15], El Sidrón 1252 from Spain [16] and Okladnikov from Russia [17].

For all datasets the statistical analyses were performed on the cloned sequences between nucleotide positions 16056–16375, with reference to the Cambridge Reference Sequence (CRS) [18]. To account for biases in the numbers of PCRs that the different datasets themselves, and different positions within each dataset, had undergone, the frequencies of the observed mutations were weighted by the number of the examined PCR at that position following [4]. For full data see Additional files 1, 2 and 3.

### Identification of hotspots

The identification of *post mortem *damage derived *hotspots *in previous studies [19,20] was through statistical comparison of the actual observed distribution against that predicted under a hypothesis of random distribution. This approach was not taken in this study due to limitations on the current Neandertal dataset (the frequency of multiple mutations takes only values 0, 1 and 2, thus a simple test of goodness of fit to a Poisson distribution of the observed pattern of mutations can not be performed). Moreover, in the previous analyses the position of the mutation itself is not considered, which is desirable if the *hotspots *themselves are to be identified. We adopted an alternative statistical procedure that enabled us to identify specific sites of *above*-expected mutation rate.

To establish the identity of *hotspots*, we initially collated the genetic information from all seven Neanderthal individuals into a single consensus individual that includes all these positions that are not polymorphic among them. Against each position in the consensus we subsequently scored the sum total number of damage-derived mutations observed (identified as described above) and the sum total number of PCRs performed over that nucleotide (see Additional file 4). The initial analytical requirement for analyzing the *hotspots *was the partitioning of the complete analyzed sequence into an equal number of bins. Adopting Sturges' rule, we therefore collapsed the 320 nucleotide sites of the alignment into 8 discreet bins containing 40 successive positions each. The expected probability of multiple mutation (per position) can therefore be calculated as $p=\frac{1}{8}\frac{\alpha }{\beta }$, where α is the total number of multiple mutations in the all region (positions 56 – 375) and β is the total number of examined PCR in the all region (positions 56 – 375). From these probabilities we obtained the expected frequencies (see Table 1) of multiple mutations per region according to the following expression: *f*_*i *_= *pn*_*i*_, where *n*_*i *_is the total number of examined PCR in region *i *(*i *= 1,..., 8). For detailed justification of the method refer to supplementary information.

**Table 1:** Summary data including observed and expected number of consistent mutations observed over the discrete HVS1 region analysed considering a Neandertal consensus sequence.

	Region								
	1	2	3	4	5	6	7	8	
	Positions (16---)								

	56–95	96–135	136–175	176–215	216–255	256–295	296–335	336–375	
Neandertal consensus HVS1									Total

Consistent mutations	9	10	13	16	10	19	1	7	85
PCR	667	597	655	734	957	724	666	583	5583
Expected mutations	1.269	1.136	1.246	1.397	1.821	1.378	1.267	1.109	

PCR refers to the number of independent cloned PCR reactions over the region.

## Results and Discussion

The nucleotide composition of the consensus Neandertal mtDNA sequence is shown in Table 2. Also indicated is the number of nucleotide positions within the dataset that are observed to contain consistent miscoding lesions of any type (i.e. prior to selection for data analysis). For the complete list of the nucleotide composition of the miscoding lesions, see Additional file 3.

**Table 2:** Consistent miscoding lesions observed among the dataset.

	A	C	G	T	
A	100		8	2	110
C	1	39		67	107
G	2		29		31
T		5		67	72
	103	44	37	136	320

Vertical: original nucleotide composition; Horizontal: observed nucleotide changes.

The fraction of the total C nucleotide positions that are observed to contain sequencing errors (63.55%) is much higher than those of A, G and T (9.09%, 6.45% and 6.94%, respectively). Of the cytosine mutations themselves, 98.5% represent C→T changes, while the only two consistent sequence modifications detected in positions containing G nucleotides are G→A changes. In light of current understandings of DNA damage, this observation of a heavy bias towards C damage is extremely odd. Due to the complementary nature of the DNA molecule, any C→T modification on a particular DNA strand within the double helix (say the mtDNA Light strand) will be manifested after PCR amplification and sequencing as either a C→T miscoding lesion on the descendent Light strand molecules, or as the complementary G→A miscoding event on the complementary strands (in this example the mtDNA Heavy strand) [21]. In contrast, any C→T damage event on a Heavy strand molecule will lead to either a C→T modification on descendent Heavy strand molecules, or G→A mutations on descendent Light strand molecules. As C→T mutations form the only credible source of DNA damage-derived miscoding lesions [8,9], a consequence of this argument is as follows. If C→T DNA damage occurs with equal probability on both Heavy and Light strand template molecules, at a frequency that is only dependent on the strands' base compositions, then the damage should be manifested as observations of both C→T and G→A sequence modifications within cloned Light strand descendent sequences, at a frequency dependent on the base composition. It is with this regard that the 7 Neandertal sequences appear striking – the observed ratio of C→T:G→A consistent sequence modifications is 67:2, a marked deviation from the approximate 3.5:1 that would be expected under the hypothesis of equal likelihood of DNA damage per different template strand (calculated as the ratio of cytosines on the Light strand:cytosines on the Heavy strand in Table 2). The implication therefore, is either that the Light strand molecule is subject to proportionally more damage-derived miscoding lesions than the Heavy strand molecule in the Neandertal datasets, or the 7 Neandertal datasets, all derived using different means by different researchers in different laboratories, all suffer from a common form of methodological bias or weakness.

**Figure 1:**
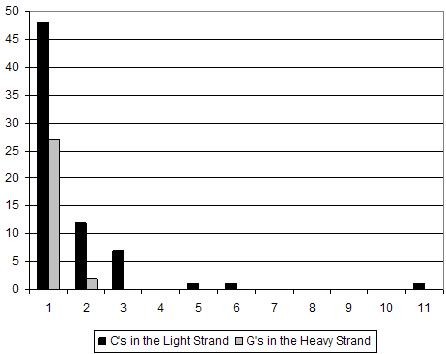
Differential clustering (from singletons to 11 repeats) of cytosines on the mitochondrial Light Strand and guanines on the Heavy Strand of the Neandertal sequences (the 16054–056 and 16375–380 clusters extend beyond the 16055–16375 studied region).

These observations are not without precedent. In a previous study on aDNA damage [19] an apparent bias of original mtDNA Light strand template molecules was observed among the data, although this could not be supported by statistical analysis. The same study also provided evidence that the Heavy strand might be subjected to increased rates of DNA degradation or damage (with respect to the Light strand) in such a way that limits PCR amplification. These observations would seem to be supported by the data observed here. While the potential cause of this is difficult to ascertain, one possible reason might be that the high levels of G and T bases in the Heavy strand somehow predispose it to PCR inhibiting damage. An alternative explanation could be the different patterning of C's in the Light strand, and of C's in the Heavy strand (Figure 1). The Light strand has 45 out to 107 cytosines (42.1%) in singletons, being the rest clustered from 2 to 12 (clusters of N = 3 C's in positions 16054–056, 16071–073, 16259–261, 16266–268, 16290–292, 16294–296, 16353–355; of N = 4 or 5 C's in 16362–366 or 16363–366, depending on the Neandertal; of N = 6 C's in 16375–380, and of N = 11 or 12 C's, depending on the Neandertal, in positions 16182–193 or 16183–193). In contrast, the Heavy strand has 27 out to 31 cytosines (87.1%) in singletons, with only two clusters of 2 cytosines (in positions 16273–274 and 16369–370). Thus, it could be that the presence of more than one cytosine increases the chances of these being damaged. A possible mechanism to explain this could be an increased G depurination (i.e. the hydrolysis of G from the deoxyribose-phosphate DNA backbone) rate in the opposite DNA strand when contiguous purines are present. The G depurination would create a nick exposing the C complementary nucleotide that could then be preferentially deaminated [8].

**Figure 2:**
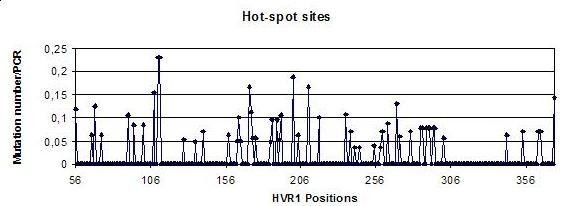
**Distribution of *hotspot *positions across the Neandertal HVS1 region**. *Hotspot *strength is measured as the ratio between observed mutations and number of independent PCRs sequenced across the position

Significant differences between observed and expected frequencies are found with regards to the existence of specific *hotspots *within the Neandertal HVS1 region ($\chi_{7}^{2}$ = 673.16, p-value = 0.000), with the largest concentration of *hotspots *being observed at positions 16108, 16111, 16112, 16172, 16201, 16211 (see Figure 2). Intriguingly, three of these positions (16108, 16111, 16112) represent controversial Neandertal specific SNPs reported in the first Neandertal sequence [11], but the authenticity of which have been questioned since [12,15]. We note that these positions are not consistent with those reported previously, either in aDNA damage or *in vivo *mutation studies. The exception is np 16172 that has been observed as hypermutable in a large number of modern studies [22]. It is noteworthy that none of these positions are placed in the 16182 (or 16183)-16193 C stretch. If our hypothesis on the C clustering is correct, it may seem that the damage in this section is underrepresented in the current published Neandertal sequences, and thus it is likely to increase in future studies. It is impossible to demonstrate the presence of *hotspots *in the HVS1 with the available data, as its existence may be extremely dependent on the underlying DNA sequence, with small differences in the sequence (for instance, in the presence of contiguous cytosines) manifesting large changes in *hotspot *distribution, but also in the number of starting template molecules in each PCR reaction, something impossible to quantify at present. However, it could be advisable to retrieve these unstable HVS1 positions at least in two independent PCRs in future studies, to prevent possible errors.

In conclusion, the possibility of comparing Neandertal PCR-generated sequence data with future sequence data derived from alternative, non-PCR based approaches (such as 454 pyrosequencing or SPEX methodology) could generate more reliable sequence data for damage analysis and could help explain the bias observed here towards C→T over G→A miscoding lesions.

## Authors' contributions

OL, CL-F and EG created the Neandertal cloning database; SV and CA analyzed the data; MTPG and CL-F wrote the paper.

## Supplementary Material

Additional file 1 Distribution of consistent mutations in each Neandertal's mtDNA. Original distribution of mutations (only consistent substitutions) and examined PCRs for each mtDNA position between 16056 and 16375. Click here for file

Additional file 2 Summary of consistent mutations. Summarized distribution of mutations and examined PCR in a prototypal individual. Click here for file

Additional file 3 Nucleotide changes for each consistent mutation. Nucleotide changes for each consistent mutation in Neandertal's mtDNA. Click here for file

Additional file 4 Statistics used for estimating the damage distribution. Calculation of the expected probability of multiple (consistent) mutations per region in the mtDNA hypervariable region 1. Click here for file
