# Supplementary material for: Statistical analysis of post mortem DNA damage-derived miscoding lesions in Neandertal mitochondrial DNA
Source: BMC Res Notes. 2008 Jul 10;1:40. doi: 10.1186/1756-0500-1-40 (PMC2547106; doi:10.1186/1756-0500-1-40)
Supplement: Additional file 4 — Statistics used for estimating the damage distribution. Calculation of the expected probability of multiple (consistent) mutations per region in the mtDNA hypervariable region 1. [file 1756-0500-1-40-S4.doc]

**Calculation of the expected probability of multiple (consistent) mutations per region in the mtDNA hypervariable region 1**.

The 320 nucleotide sites of the Neandertal mtDNA HVR1 alignment are collapsed into 8 discreet bins containing 40 successive positions each.

P(multiple mutations in the total region, i.e. positions 56 – 375)

= $\sum_{\text{i}=1}^{8} \text{P(multiple mutations in region i)}$

as we consider the hypothesis that these probabilities are equal (there are no hot-spot positional)

$$\text{P(multiple mutations in region i)}$$

= 1/8 P(multiple mutations in the total region, i.e. positions 56 – 375)

and

P(multiple mutations in the total region, i.e. positions 56 – 375)

= $\sum_{j=1}^{320} P(\text{we are in position }j\text{)}P(\text{multiple mutations/we are in position }j)$

= $\sum_{j=1}^{320} \frac{\text{examined PCR for position }j}{\text{total examined PCR}}\times\frac{\text{total multiple mutations for position }j}{\text{examined PCR for position }j}$

= $\sum_{j=1}^{320} \frac{\text{total multiple mutations for position }j}{\text{total examined PCR}}$

= $\frac{\text{total multiple mutations }}{\text{total examined PCR}}$

Finally, $\text{P(multiple mutations in region i)}$

= 1/8$\frac{\text{total multiple mutations }}{\text{total examined PCR}}$.

.
